# Pectic Polysaccharides from Dragon Fruit Peel: Structure-Function Relationships and Emerging Potential in Synbiotic Food Systems

**DOI:** 10.3390/foods15122073

**Published:** 2026-06-08

**Authors:** Dumila Roshani, Zeqian Yang, Zixin Han, Nan Shang

**Affiliations:** 1 College of Engineering, China Agricultural University, Beijing 100083, China; dumilaroshani@cau.edu.cn (D.R.); hzx919@cau.edu.cn (Z.H.); 2 College of Food Science and Nutritional Engineering, China Agricultural University, Beijing 100083, China; s20233061185@cau.edu.cn; 3 Food Laboratory of Zhongyuan, Luohe 462000, China

**Keywords:** agro-industrial waste, calcium crosslinking, circular bioeconomy, dietary fiber, functional foods, gut microbiota modulation, probiotic delivery, rhamnogalacturonan-I, waste valorization

## Abstract

The valorization of agro-industrial byproducts is attracting attention due to its potential to support circular bioeconomy development in food systems. Dragon fruit (*Selenicereus* spp.) peel, representing approximately one-third of total fruit mass, is an underutilized biomass that is high in pectin content. Unlike standardized commercial citrus and apple pectins, pectin from dragon fruit peel exhibit variability in their galacturonic acid content, degree of esterification, molecular weight, and rhamnogalacturonan-I branching structure, which are dependent on how the pectin is extracted. These structural attributes influence the solubility, rheological properties, gelation mechanisms, emulsifying capacity, and water-holding properties. There is emerging evidence that rhamnogalacturonan-I-enriched fractions promote the growth of beneficial microorganisms and may also increase the in vitro production of short-chain fatty acid, thereby exhibiting potential prebiotic activity. In addition, low methoxyl pectin has been shown to provide excellent properties for the calcium-mediated encapsulation of probiotics, as well as for pH-sensitive release in the gastrointestinal tract, thus supporting the synbiotic concept. The purpose of the current paper is to provide an overview of recent findings related to extraction technologies, structural characterization, structure–function relationship, fermentation behavior, potential delivery of probiotics, and the regulatory requirements for using dragon fruit peel pectin in the development of functional foods.

## 1. Introduction

Concerns about the accumulation of agro-industrial waste and its effects on the environment have grown due to the rapid expansion of the world’s fruit processing industries [1,2]. Fruit peels, pomace, and seeds are produced in large quantities each year and are frequently underutilized despite being rich sources of dietary fibers and bioactive compounds [3,4]. As part of circular bioeconomy initiatives to transform food byproducts into high-value functional ingredients, the idea of waste valorization has garnered significant interest in recent years [5,6]. In this context, fruit peel-derived polysaccharides, especially pectin, have shown promise as ingredients in functional foods that promote gut and metabolic health [7,8,9].

Consumer interest in and market activity for gut health-orientated functional foods, such as those containing probiotics and prebiotics, has grown in recent years in tandem with sustainability initiatives [10,11]. It is becoming more widely accepted that the gastrointestinal (GI) tract (and its resident microbiota) plays a crucial role in controlling immunological response and metabolic balance, which has consequences for overall health. As a result, probiotic, prebiotic, and synbiotic system research and commercial development have intensified [12,13]. Prebiotics are substrates that host bacteria specifically use to provide health benefits, whereas probiotics are living microbes that, when given in sufficient proportions, provide health benefits to the host [14]. Pectins are particularly appealing among dietary fibers because of their complex structure, capacity to be fermented by the gut microbiota, and techno-functional adaptability in food systems (e.g., gelation, viscosity, water-holding capacity, and emulsifying behavior) [15,16]. Pectin structural characteristics, particularly degree of esterification (DE), molecular weight (MW), and branching/domain architecture, have been shown in recent studies to have a significant impact on solubility, gel/rheological behavior, fermentation kinetics, and selective modulation of gut microbial composition [17,18,19,20]. Concurrently, pectin-based matrices have drawn interest as probiotic encapsulation carriers because of their pH-responsive swelling behavior under GI conditions, gel-forming capacity in the presence of calcium ions, and biocompatibility [21,22].

Apple pomace and citrus peel have traditionally been the principal industrial raw materials used to extract commercial pectin [23]. However, due to concerns about sustainability, the value-adding of agro-industrial byproducts, and the pursuit of structurally unique pectins with unique functional characteristics, there has been a growing focus on unconventional sources [24]. Dragon fruit (pitaya) belongs to the genus *Selenicereus* (syn. *Hylocereus*) and is originally from Central and South America, but is now extensively grown across Southeast Asia, Latin America, and the Mediterranean regions of Europe [25,26]. The peel, which makes up around 30% of the fruit bulk after industrial processing, is mostly thrown away as trash. Significantly, monosaccharide and structural investigations have shown that dragon fruit peel is rich in cell wall polysaccharides, especially pectins that contain both homogalacturonan (HG)-dominant and rhamnogalacturonan-I (RG-I)-rich areas [27,28]. The peel has significant amounts of phenolic chemicals and betalain pigments, particularly betacyanins, in addition to pectic polysaccharides, which may support antioxidant activity [29]. Additionally, in vitro fermentation models have linked RG-I-enriched fractions to increased synthesis of short-chain fatty acids (SCFAs) and selective activation of favorable probiotic genera [27,30]. These results establish dragon fruit peel pectin as a possible prebiotic substrate as well as a techno-functional ingredient.

Despite the expanding literature on extraction, structural characterization, and individual techno-functional/bioactive properties of dragon fruit peel pectins, integration into system-level synbiotic design remains limited. Recent studies commonly focus on extraction-induced structural metrics (e.g., DE changes) and then assess selected isolated outcomes such as emulsifying performance, film functionality, or antioxidant activity, rather than connecting structural variation to downstream food-matrix performance and GI/microbiome-relevant endpoints in a unified workflow [29,31]. In contrast, general synbiotic and co-encapsulation studies have proposed frameworks linking matrix selection, crosslinking, and GI-triggered release [32,33,34]. However, it should be noted that these studies are not specific to dragon fruit peel pectin, and comparable dragon fruit peel pectin-specific investigations remain limited.

Therefore, this review aims to bridge that gap by systematically examining dragon fruit peel pectins from feedstock to synbiotic system design. We synthesize recent findings on extraction technologies, structural features, physicochemical properties, fermentation behavior, and potential roles in probiotic delivery. By integrating structure–function evidence with food matrix and GI conditions, this review proposes a system-level perspective for designing dragon fruit peel pectin-based synbiotic ingredients suitable for functional food applications.

## 2. Dragon Fruit Peel as a Pectin Feedstock

### 2.1. Botanical Origin and Processing-Relevant Characteristics

The red and white-fleshed cultivars of *Selenicereus* (syn. *Hylocereus*), which are currently extensively grown in tropical and subtropical production systems, are the main source of dragon fruit peel [25]. Despite being recognized as a biomass source rich in pectin, its suitability as an extraction substrate is determined by structural organization, processing history, and composition [35,36]. Dragon fruit peel pectin processes usually do not require specific de-oiling steps because dragon fruit peel-derived matrices are often low in total lipids when compared to citrus peel (essential oil-rich) [37,38]. However, depending on pH, temperature, and extraction time, the matrices closely linked to betalain pigments and phenolic chemicals may continue to be partially co-extracted [39,40]. Pectin yield, molecular weight preservation, and esterification degree can all be affected by pre-treatment factors, such as drying temperature, particle size reduction, and storage stability. As a result, upstream processing is a significant factor in determining the structural results documented in the literature [41,42,43].

### 2.2. Cell Wall Components and the Occurrence of Pectin in Dragon Fruit Peel

Dragon fruit peel’s cell wall architecture, which consists of cellulose microfibrils embedded within hemicellulosic and pectic networks, is similar to that of other fleshy fruits [44]. According to monosaccharide content and structural investigations, extracted dragon fruit pectins usually contain both HG regions and RG-I domains. Galacturonic acid (GalA) is frequently reported as the predominant residue (ranging from roughly 39% to over 87% of total monosaccharides, depending on the extraction technique and level of purification), accompanied by detectable rhamnose (Rha), arabinose (Ara), and galactose (Gal), indicating branched RG-I architecture [27,28,31,45,46]. This variation in GalA content is closely associated with extraction severity and purification strategy. Acid-based extraction and subsequent alcohol precipitation tend to enrich HG-rich fractions through selective solubilization of protopectin and partial removal of neutral sugar side chains, thereby increasing the relative GalA proportion [27,31,46]. In contrast, milder techniques such as ultrasound-assisted extraction (UAE) or non-methylesterified pectin recovery better preserve RG-I domains and associated neutral sugars, resulting in comparatively lower GalA content [28,45]. Additionally, purification steps such as dialysis or repeated precipitation can further concentrate HG regions by removing co-extracted impurities, contributing to higher reported GalA values [27,31].

RG-I side chain abundance in dragon fruit peel pectins is commonly estimated using neutral sugar ratios, namely (Ara + Gal)/Rha and associated indices [47]. Reported variations in these ratios across studies suggest that the relative distribution of HG and RG-I domains appears to be sensitive to extraction severity and solvent conditions [45,48]. The relative recovery of HG and RG-I domains is influenced by the severity of extraction, with structural implications covered in Section 3.3. Higher MW distributions and neutral sugar-rich side chains are preserved by cellulase-assisted techniques [49].

Betalain colors and phenolic chemicals may bind to the pectin matrix in addition to structural polysaccharides [50]. Numerous investigations have found quantifiable phenolic content and residual pigment fractions in extracted dragon fruit pectins, which influence color characteristics and contribute to antioxidant activity. Interfacial characteristics in emulsified systems may also be impacted by these co-extracted elements [51,52]. Therefore, instead of being a completely decolorized HG polymer like highly refined commercial pectins, dragon fruit peel pectin should be considered a composite matrix [39].

### 2.3. Comparison with Conventional Industrial Pectin Sources

Commercial pectin production is dominated by citrus peel and apple pomace, yielding food-grade pectins with high GalA contents and tightly controlled DE and MW [53,54]. These products are classified as high-methoxyl (HM; DE > 50%) or low-methoxyl (LM; DE < 50%) [55]. HM pectins gel in high-solid, low-pH systems, whereas LM pectins gel via Ca^2+^-mediated crosslinking described by the “egg-box” model [56]. Such standardized attributes underpin their reproducible performance in confectionery, dairy, and fruit-based preparations [16].

Dragon fruit peel pectins show extraction-dependent variability in DE and MW [27]. DE values range from 30 to 60% (LM to low-HM) [31,57,58], with MW from 33 kDa to >1000 kDa. This reflects extraction-induced de-esterification, depolymerization, and selective enrichment of RG-I-rich fractions [27]. Dragon fruit peel pectin’s structural variability limits direct substitution for citrus pectin in jam/confectionery but enables the creation of edible films, emulsification, and composites [31,36,59,60]. Unlike commercial pectins, it lacks industrial grading standards, positioning it as an extraction-tunable food waste polysaccharide [61,62]. A comparative overview of the compositional ranges and physicochemical characteristics of dragon fruit peel pectin relative to conventional citrus and apple pectins is presented in Table 1.

## 3. Extraction and Purification Technologies

### 3.1. Conventional Extraction Approaches

Acid extraction is the dominant conventional route for pectin recovery from fruit peels. A widely cited extraction description is an acidic aqueous medium at pH ~1.5–3, typically ~75–100 °C, for ~1–3 h, which solubilizes pectin via acid hydrolysis of insoluble pectic substances/protopectin [24,64]. In dragon fruit peel specifically, hot-acid extraction has been used to obtain pectin and evaluate structure–property relationships (including comparisons to commercial pectins), confirming that dragon fruit is a viable pectin source under conventional acidic processing [31]. Because extraction intensity alters pectin chemistry, conventional acid conditions are best treated as a structural “tuning” step rather than a neutral recovery method: acid/heat severity is associated with changes in esterification and chain integrity (demethylation and hydrolytic cleavage), which shifts molecular size distributions and related functional attributes. Specifically, extraction under low pH and high temperature promotes demethylation of GalA residues, resulting in reduced DE, while prolonged heating and acidic conditions induce hydrolytic cleavage of glycosidic bonds, leading to a decrease in MW. Conversely, milder conditions or shorter extraction times better preserve ester groups and polymer chain integrity, resulting in higher DE and MW [64,65]. For dragon fruit peel pectin, a recent dragon fruit-focused study reported that extracted pectin can span LM classifications, with DE and average MW varying widely across preparations (DE 8.51–50.64% and MW 115.23–577.84 kDa) [66].

### 3.2. Green and Emerging Extraction Technologies

Emerging extraction strategies are being explored to improve sustainability and structural control in dragon fruit peel pectin recovery (Table 2). In comparison to conventional extraction in dragon fruit peel systems, UAE has been studied in relation to dragon fruit peels and has been demonstrated to improve extraction performance. This is consistent with the UAE’s cavitation-driven enhancement of mass transfer as reported in more general extraction literature [28,48,67]. Dragon fruit peel pectin has also been extracted using microwave-assisted extraction (MAE); studies report higher extraction yields when compared to conventional heating, but they also point out that excessive microwave intensity and prolonged extraction time can lead to pectin degradation, including reductions in MW and viscosity due to polymer chain cleavage. Such degradation can be detected through decreases in intrinsic viscosity, changes in MW distribution (e.g., Size Exclusion Chromatography [SEC] analysis), or alterations in rheological properties [68,69]. For red dragon fruit peel pectin, subcritical water extraction (SWE/SCWE) has been reported as a more effective, acid-minimized method that can also change the esterification properties of the recovered pectin fraction [66]. For greener solvent systems, Deep Eutectic Solvents/Natural Deep Eutectic Solvents (DESs/NADESs) are being more commonly discussed as alternatives for pectin extraction across fruit wastes; however, dragon fruit peel-specific DES/NADES pectin studies remain comparatively limited [65,70].

### 3.3. Extraction-Dependent Structural Modulation

For dragon fruit peel pectin, extraction method is consistently linked to differences in recovered pectin structure and functionality. These differences arise from distinct physicochemical mechanisms: acidic and high-temperature conditions promote hydrolysis of protopectin and demethylation of GalA residues, leading to reduced DE, while severe thermal or microwave treatments induce depolymerization through glycosidic bond cleavage, resulting in lower MW. In contrast, milder or non-thermal processes tend to preserve side chain structures, particularly RG-I regions, resulting in more branched and higher-MW pectins [67,72]. Dragon fruit processing by UAE indicates that extraction can yield non-/low-methylesterified pectin with distinct molecular features, underscoring that intensified extraction can change esterification-related classification and polymer characteristics [28]. MAE studies on dragon fruit peel further show that microwave power/time materially affects extraction outcome, with reports of higher yield under stronger MAE conditions but also indications of possible degradation under more intense processing, highlighting a practical trade-off between recovery and structural preservation [68,69]. Likewise, SCWE of red dragon fruit peel pectin has been reported to alter key properties (including esterification behavior) and to produce pectin suitable for downstream material applications (e.g., edible films), reinforcing that extraction choice functions as a structural design lever, not just a recovery step [66]. In parallel, broader pectin-processing reviews support that processing intensity (thermal/chemical/mechanical) governs demethylation and depolymerization tendencies, helping to interpret why dragon fruit peel pectin properties vary among extraction routes and conditions [67,72].

### 3.4. Extraction Yield and Its Relationship with Structure

Extraction yield is an important parameter in evaluating the practical applicability of dragon fruit peel pectin, particularly in relation to process efficiency and potential industrial utilization. It has been reported that the yield of dragon fruit peel pectin varies considerably depending on the extraction method, solvent system, and processing conditions, with values typically reported in the range of approximately 9% to 16% on a dry weight basis [66]. For comparison, commercial citrus and apple pectins typically exhibit higher extraction yields, commonly reported in the range of approximately 15% to 30% depending on raw material characteristics and processing conditions [7,73]; however, dragon fruit peel pectin, despite its comparatively moderate yield, offers distinct structural features and functional potential, highlighting the importance of optimizing extraction conditions to balance yield and functionality.

In general, acid-assisted extraction methods, including the use of citric or mineral acids, tend to produce higher yields due to enhanced solubilization of HG regions and disruption of the plant cell wall matrix [66]. However, it should be noted that such conditions are often associated with partial depolymerization and modification of structural features, including reductions in MW and changes in the DE, which may influence functional performance [74]. In contrast, milder approaches such as hot water extraction typically result in lower yields but are more effective in preserving native structural features, particularly RG-I domains and side chain integrity [66].

It has also been reported that emerging extraction techniques, such as ultrasound-assisted and enzyme-assisted methods, may improve extraction efficiency while maintaining structural characteristics, although the reported yields remain dependent on processing parameters and raw material variability [74]. Overall, these observations suggest that extraction yield is closely linked to structural outcomes, and therefore represents a key factor in balancing extraction efficiency and functional performance in dragon fruit peel pectin systems.

## 4. Structural Features of Dragon Fruit Peel Pectins

### 4.1. Pectic Domain Architecture: HG, RG-I, and RG-II

HG, RG-I, and RG-II are the three primary polysaccharide domains of plant cell wall pectins. Partially methyl-esterified and perhaps acetylated α-(1 → 4)-linked GalA residues comprise the linear backbone of HG. RG-I is composed of a repeating [→ 4)-α-D-GalA-(1 → 2)-α-L-Rha-(1 →] backbone with Ara- and Gal-rich side chains attached to Rha residues [75,76]. RG-II is a conserved, low-abundance domain capable of forming borate diester crosslinks contributing to cell wall integrity [77,78]. Structural analyses of dragon fruit peel pectins consistently indicate the co-presence of HG and RG-I domains, inferred from monosaccharide profiles rich in GalA together with Rha, Ara, and Gal. The relative proportion of these domains is extraction-dependent, influencing the esterification degree and MW [31,79].

### 4.2. Monosaccharide Composition

GalA is consistently reported as the predominant monosaccharide in dragon fruit peel pectin, reflecting the dominance of an HG-rich backbone. Variations in GalA proportion across studies reflect differences in extraction strategy and severity, as discussed in Section 3.3 [31]. Rha, Ara, and Gal are consistently detected as secondary residues and collectively serve as markers of RG-I domain abundance and side chain complexity. Rha content is generally reported in the range of 1–7% and Ara in the range of 3–15%, with Gal varying from approximately 3–12%. Xylose, mannose, and glucose are typically detected in trace amounts and are attributed to co-extracted hemicellulosic or starch-derived material rather than the pectic backbone itself. The molar ratios of these neutral sugars, in particular the (Ara + Gal)/Rha index, are routinely used in dragon fruit studies as a proxy for RG-I side chain density [27,80,81].

### 4.3. DE and Acetylation

DE is the most widely reported structural parameter in dragon fruit peel pectin studies and is extraction-sensitive, as acid/heat and alkali/enzyme treatments selectively demethylate the HG backbone. Reported DE values for dragon fruit peel pectins span a broad range from below 10% in highly de-esterified preparations (e.g., subcritical water or prolonged acid treatment) to over 65% in milder extractions, but the majority fall in the LM to borderline HM range of approximately 30–60%. This makes dragon fruit peel pectins structurally distinct from tightly controlled HM citrus pectins and LM commercial grades [27,28,45,57].

Acetylation (O-acetyl substitution at C-2 or C-3 of GalA residues) has been less systematically characterized in dragon fruit peel pectins than DE, with inconsistent or absent reporting across studies representing a key structural gap. Low acetyl contents are typical in fruit pectins (often <5%), where even minor levels influence gel texture, water retention, and enzymatic susceptibility, though dragon fruit-specific values remain unreported. Future work should prioritize acetylation quantification via Proton Nuclear Magnetic Resonance Spectroscopy (1H-NMR) or High-Pressure Liquid Chromatography (HPLC) to address this omission [75,82].

### 4.4. MW Distribution

Dragon fruit peel pectins’ MW statistics show some of the largest known ranges of any non-conventional pectin source, with weight-average MW values ranging from about 33 kDa to values over 1000 kDa. This range reflects true extraction-dependent variation rather than just methodological inconsistency: acid conditions at high temperatures gradually depolymerize the pectin backbone to 33.52 kDa, while enzyme-assisted, UAE, or lower-temperature routes better preserve chain integrity up to 645–1181 kDa. Values suggest heterogeneous domain structure (HG + RG-I) without industrial fractionation steps when reported with dispersity indices (polydisperse, polydispersity index [PDI] > 2) [28,29,31].

Most studies report MW as a single weight-average value determined by SEC without Multi-Angle Light Scattering (MALS) correction, which can introduce systematic overestimation due to the non-spherical, rod-like conformation of pectic chains in solution. Only a subset of dragon fruit-focused studies employ SEC-MALS, providing conformation-independent MW; this methodological variation limits direct cross-study comparison and is reflected in the ranges consolidated in Table 3 [52].

## 5. Structure–Function Relationships in Dragon Fruit Peel Pectins

### 5.1. Solubility and Charge Characteristics

**Figure 1:**
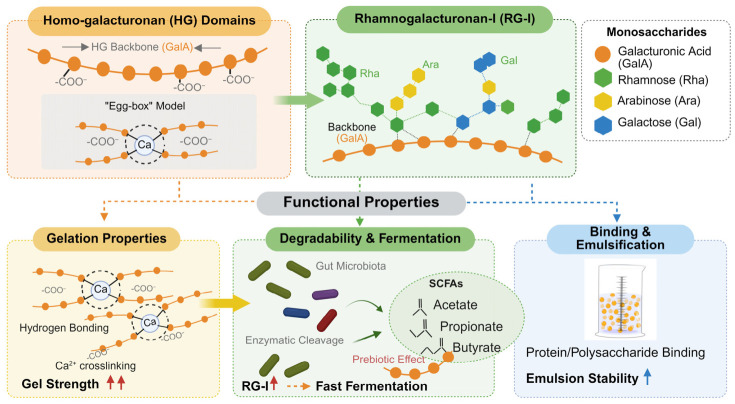
Pectin structure–function relationship.

The solubility of dragon fruit peel pectins is closely associated with DE, which determines backbone charge density. Lower DE increases the proportion of ionizable carboxyl groups, enhancing electrostatic repulsion between polymer chains and improving aqueous dispersibility, particularly at moderate-to-neutral pH (≈pH 4–7) [45,74,83]. In contrast, HM pectins (DE > 50%) exhibit reduced solubility under acidic conditions (typically pH < 3.5), where protonation of carboxyl groups decreases charge repulsion and promotes aggregation, while LM pectins (DE < 50%) remain more soluble across pH ≈ 3–7 due to their higher charge density. Dragon fruit peel pectins typically exhibit intermediate DE values (30–60%), occupying a charge-intermediate zone that provides better solubility than HM pectins (>65% DE) in high-solute food matrices while avoiding the excessive ionization of fully de-esterified LM pectins [84]. In less-purified fractions obtained via ultrasound or NADES extraction, co-extracted phenolics and betalains form non-covalent polymer–polyphenol associations that modulate apparent solubility [58]. Figure 1 depicts the structure–function relationships of dragon fruit peel pectin.

### 5.2. Viscosity and Rheological Behavior

Solution viscosity correlates directly with MW in dragon fruit peel pectins. Comparative extraction studies show cold water- and hot water-extracted fractions (MW 1010–1210 kDa) exhibit substantially higher apparent viscosity across shear rates compared to enzymatic preparations (~840 kDa), with all dragon fruit pectin solutions displaying pseudoplastic (shear-thinning) behavior (*n* < 1.0) [66]. MW remains the primary determinant of flow resistance in dilute and semi-dilute regimes due to chain entanglement. In addition to MW, DE also influences rheological behavior. LM pectins (DE < 50%) generally exhibit lower viscosity at equivalent MW due to increased electrostatic repulsion and more expanded chain conformations, whereas HM pectins (DE > 50%) tend to form more compact structures and can exhibit higher apparent viscosity under acidic and high-solid conditions due to reduced charge repulsion and enhanced hydrophobic interactions [24]. RG-I side chains amplify solution viscosity by increasing hydrodynamic radius [85].

### 5.3. Gelation

The gelation mechanism is primarily determined by DE. LM pectins (DE < 50%) form thermostable Ca^2+^-crosslinked “egg-box” junction zones across pH 2–6, whereas HM pectins (DE > 50%) require acidic conditions (pH < 3.5) and >55% soluble solids [23]. Dragon fruit peel pectins exhibit borderline DE values (46–52%) that enable access to either gelation mechanism depending on Ca^2+^ availability and pH [28]. Ca^2+^-induced gel strength depends on the degree of blockiness (DB) of contiguous GalA sequences [86]. However, DB has not been systematically reported for dragon fruit peel pectin preparations; this structural gap and its implications for structure–function predictability are discussed in Section 11.1.

### 5.4. Emulsification

The emulsifying performance of dragon fruit peel pectin reflects the combined effects of MW and structural heterogeneity. Higher-MW preparations generally form thicker interfacial adsorption layers and show improved emulsion stability [27]. Dragon fruit peel pectin has been reported to outperform certain commercial pectins in oil-in-water systems, suggesting that compositional complexity contributes to functionality [31]. Associated non-carbohydrate components, including residual proteins or phenolics, may further enhance interfacial interactions, although their quantitative contribution in dragon fruit systems requires further clarification [69].

### 5.5. Water-Holding and Oil-Holding Capacity

The water-holding capacity (WHC) for dragon fruit peel pectins ranges from approximately 4 to 7.5 g water/g dry sample, with the highest values associated with enzyme-assisted extractions that preserve branched RG-I side chain architecture [27,41,57]. Lower DE and higher RG-I branching density create open, hydrated polymer networks that resist self-association and maximize the water-accessible surface area [23,24]. MW contributes secondarily through network extensiveness [24]. The oil-holding capacity (OHC) (~2 g oil/g) is substantially lower, as expected for hydrophilic polysaccharides, and is more sensitive to methyl ester content (DE) and co-extracted hydrophobic material than to branching. Moderate-DE, less-purified dragon fruit peel pectins therefore outperform highly purified LM fractions in oil-binding applications, while the reverse applies for water-retentive encapsulation matrices [41,57]. Table 4 summarizes functional properties related to dragon fruit peel pectin with their primary structural drivers and mechanisms.

## 6. Food-System Applications of Dragon Fruit Peel Pectins

### 6.1. Dairy: Yogurt and Fermented Beverages

By preventing whey syneresis and preserving viscosity during storage, pectin stabilizes fermented dairy products through electrostatic interactions between its anionic carboxyl groups and positively charged casein micelles at pH < 5.0. Compared to HM pectins, lower DE pectins have a higher charge density and form stronger connections with casein that improve gel cohesiveness and WHC [24]. Red dragon fruit peel has been successfully incorporated into yogurt formulations. Fortification with 2–7% peel powder improves antioxidant properties, dietary fiber content, and sensory acceptability while supporting probiotic viability [87]. Separate synbiotic yogurt studies using peel extract with probiotic cultures (e.g., *L. bulgaricus*, *S. thermophilus*) demonstrate prebiotic potential from pectin and fiber fractions, promoting bacterial growth during fermentation. Fruit pulp incorporation reviews affirm such systems as synbiotic when combining live cultures with fermentable plant fibers [88]. In dairy systems, LM pectins reduce excessive acidification and enhance WHC relative to HM pectins, which increase gel hardness and sourness perception; these effects are well-documented for citrus/apple pectins but remain extrapolations for dragon fruit peel varieties. While dragon fruit peel enhances yogurt functionality, direct evidence of its pectin stabilizing casein in acidified beverages (pH < 4.5) is currently limited, with research focusing primarily on yogurt fortification rather than beverage applications [89].

### 6.2. Beverages and Emulsified Systems

Pectin serves as a cloud stabilizer, colloid dispersant, and mouthfeel enhancer in beverages like fruit juices and high-protein drinks, with functionality driven by MW for viscosity/steric stabilization and charge density (DE-dependent) for electrostatic interactions with suspended particles [24,90,91]. Dragon fruit peel pectin from SCWE yields LM pectin (DE 8.5–50.6%, MW 115–578 kDa) suitable for colloidal stabilization. The material demonstrates emulsifying capacity > 70% for cold-/hot-water extracts, outperforming lower-MW fractions and positioning it for high-protein or plant-based beverages requiring oil–protein–polysaccharide interface stabilization. Less-purified extracts retain co-extracted phenolics and betalains, providing antioxidant activity that could protect emulsified systems during storage, as evidenced in analogous pectin-based emulsions [52,66,91]. Direct beverage applications remain exploratory, but high-MW dragon fruit pectin from gentle extractions shows promise for preventing sedimentation and phase separation, consistent with general pectin beverage stabilization mechanisms [90].

### 6.3. Structured Foods and Gels

Many reported dragon fruit peel pectins fall in a borderline DE window (~46.8–51.8%), which places them near the transition between LM-type Ca^2+^ gelation and HM-type sugar–acid gelation, potentially widening formulation flexibility compared with single-mechanism commercial grades [27,92]. In general, Ca^2+^-mediated “egg-box” junctions depend on the availability and DB of non-esterified GalA residues, so gelation behavior is strongly governed by DE and the distribution of free carboxyl groups [55]. For structured textures and emerging fabrication uses, LM pectin hydrogels are established as printable/shape-stable food inks because they undergo ionotropic gelation under mild Ca^2+^ conditions with tunable rheology [55,92]. SCWE-derived dragon fruit peel pectin forms cohesive polymer networks suitable for structured matrices, indicating its potential for gel-based and textural food applications [66]. For conventional fruit spreads, dragon fruit peel pectin has been successfully applied in jam systems, although some studies report that relatively high pectin levels may be required to achieve a firm set compared with standardized commercial pectins, implying a need for formulation optimization [57].

### 6.4. Edible Films and Active Packaging

Edible film formation represents the most extensively investigated food-system application of dragon fruit peel pectin in the recent literature. Film performance is governed primarily by MW, DE, plasticizer content, and intermolecular interactions, which collectively determine tensile strength, flexibility, thermal behavior, and moisture permeability [55]. In a direct comparison of extraction methods, Tristanto et al. (2024) [66] reported that acid-extracted dragon fruit peel pectin films exhibited superior mechanical performance (tensile strength 2.12–4.11 MPa; elongation at break 48.72–61.39%), whereas SCWE films showed enhanced thermal stability (Tdmax ≈ 250 °C) and lower water vapor permeability (5.59 × 10^−11^ g·cm^−1^·s^−1^·Pa^−1^). These findings illustrate the structural trade-offs associated with extraction-induced variations in MW and DE. Beyond standalone films, dragon fruit peel pectin has also been applied in biocomposite films containing peel extracts, where co-extracted phenolics/pigments contribute antioxidant activity [93], and, in related systems incorporating dragon fruit peel anthocyanins/betacyanins, colorimetric responsiveness suitable for freshness indication has been demonstrated [94,95]. Blending and crosslinking strategies, particularly with chitosan, can enhance the mechanical and barrier performance (and often the antimicrobial functionality) of pectin-based films [96]. However, chemical crosslinkers such as glutaraldehyde raise food-safety concerns, and variability in pectin structure necessitates standardization before commercial translation [55,97].

## 7. Prebiotic Potential and Fermentation Outcomes of Dragon Fruit Peel Pectins

### 7.1. Digestive Resistance and Colonic Availability

Pectins are generally resistant to digestion in the upper GI tract due to the absence of endogenous pectinolytic enzymes in humans, allowing them to reach the colon where they are subsequently fermented by the gut microbiota [98,99,100,101]. While this behavior has been extensively reported for commercial citrus and apple pectins, it should be noted that direct experimental evidence describing the digestion behavior of dragon fruit peel pectin is still limited. Under simulated GI conditions, pectins may undergo partial structural modifications, including reductions in MW and changes in the degree of esterification, depending on their initial structural characteristics and surrounding matrix [100,101]. These changes do not eliminate fermentability but may influence physicochemical properties prior to colonic exposure. However, current understanding of dragon fruit peel pectin digestion is largely inferred from general pectin studies, and its specific digestion kinetics and structural transitions remain insufficiently characterized.

### 7.2. Structure-Dependent Microbial Fermentation

The fermentation of pectin is mediated by gut microbiota capable of degrading both HG and RG-I domains, with structural features such as DE and side chain composition influencing microbial utilization [99,102]. In the case of dragon fruit peel pectin, it has been reported that RG-I-rich fractions are more readily utilized by beneficial microorganisms, highlighting the importance of neutral sugar side chains in fermentation behavior. For example, RG-I-enriched dragon fruit peel pectin fractions have been shown to promote the growth of *Bifidobacterium animalis* subsp. *lactis*, suggesting that Ara- and Gal-rich side chains may contribute to selective microbial fermentation [47].

However, it should be emphasized that most of the available evidence is derived from *in vitro* and single-strain studies, and comprehensive investigations involving complex gut microbial communities are still lacking. A more detailed discussion of dragon fruit peel pectin–probiotic interactions is provided in Section 7.4.

### 7.3. SCFA Production and Microbial Metabolites

SCFAs, including acetate, propionate, and butyrate, are the primary metabolites produced during pectin fermentation, with acetate commonly reported as the dominant product [99,103]. It has been reported that SCFA production is influenced by both the structural characteristics of pectin and the composition of the microbial community involved in fermentation [102].

In the context of dragon fruit peel pectin, SCFA production has not been extensively quantified, and most conclusions are inferred from microbial growth studies rather than direct metabolite analysis [61]. Available evidence suggests that RG-I-rich dragon fruit peel pectin fractions may enhance the proliferation of beneficial bacteria, which are associated with SCFA generation [30]; however, direct measurements of SCFA profiles resulting from dragon fruit peel pectin fermentation remain scarce [47]. Therefore, although dragon fruit peel pectin is expected to contribute to SCFA production based on its structural similarity to other pectins [98], further experimental validation is required to establish clear relationships between dragon fruit peel pectin structure and specific fermentation outcomes.

### 7.4. Dragon Fruit Peel Pectin and Probiotic Growth

It has been reported that dragon fruit peel pectin can support probiotic growth, particularly through RG-I-enriched fractions. Comparative studies have demonstrated that citric acid-extracted dragon fruit peel pectin, characterized by higher RG-I content and increased neutral sugar side chain density, promotes greater proliferation of *Bifidobacterium animalis* subsp. *lactis* compared to hot water- or ultrasound-extracted fractions [47].

Further structural investigations have indicated that RG-I domains, especially galactan-rich side chains, play a key role in enhancing probiotic growth, likely by serving as accessible substrates for microbial metabolism [9]. These findings support a structure–function relationship between dragon fruit peel pectin composition and its bifidogenic potential.

In addition, clinical evidence from dragon fruit-derived oligosaccharides (DFOs) (although not derived from intact dragon fruit peel pectin) has demonstrated increased *Bifidobacterium* abundance and improved immune-related markers in human subjects, suggesting that dragon fruit polysaccharides possess measurable prebiotic effects [104]. However, it should be noted that direct *in vivo* evidence specifically for intact dragon fruit peel pectin remains limited, and most available studies are restricted to *in vitro* or fraction-based systems. Current dragon fruit peel pectin evidence primarily derives from single-strain or fraction-based studies (Table 5).

## 8. Dragon Fruit Peel Pectin for Probiotic Delivery

### 8.1. Need for Protective Delivery Systems

Probiotic microorganisms are sensitive to environmental stresses encountered during processing, storage, and GI transit, including low pH, bile salts, and enzymatic activity [106,107,108]. These conditions can significantly reduce cell viability before reaching the colon and therefore highlight the need for protective delivery systems.

### 8.2. What Pectin Matrices Can Do: General Evidence

In general, pectin-based delivery systems can be broadly classified into LM and HM types, which differ in their gelation mechanisms and applicability in encapsulation systems. LM pectins form Ca^2+^-mediated ionotropic hydrogels under mild conditions, making them particularly suitable for probiotic encapsulation. In contrast, HM pectins require high sugar concentrations and low pH to form gels, conditions that are less favorable for maintaining probiotic viability. Therefore, LM-type pectins are more commonly investigated for probiotic delivery applications, whereas HM pectins are more frequently used in conventional food gel systems [74,107,108]. In the case of dragon fruit peel pectin, it has been reported that they can function as a wall matrix for the encapsulation of bioactive compounds, particularly betalains and phenolic compounds. For example, dragon fruit peel pectin has been applied in coacervation and freeze-drying systems, achieving encapsulation efficiencies ranging from approximately 60 to 93% for pigment stabilization under varying environmental conditions [40]. These findings suggest that dragon fruit peel pectin can form matrices suitable for encapsulation purposes.

However, it should be noted that most available studies focus on the encapsulation of small bioactive molecules rather than live probiotic cells. As a result, although dragon fruit peel pectin has demonstrated promising matrix-forming properties, its direct application in probiotic encapsulation systems remains insufficiently explored.

### 8.3. Current Evidence for Dragon Fruit Peel Pectin

At present, experimental evidence evaluating probiotic viability within dragon fruit peel pectin-based systems under simulated GI conditions is still limited. Most studies on pectin-based probiotic delivery systems are based on commercial citrus or apple pectins, and these findings cannot be directly extrapolated to dragon fruit peel pectin due to its distinct structural characteristics [107]. Furthermore, key parameters such as release kinetics, gel stability, and cell survival during digestion have not been systematically investigated for dragon fruit peel pectin.

### 8.4. Implications for Dragon Fruit Peel Pectin as a Delivery Matrix

Based on its structural features, including moderate DE and RG-I-rich architecture, dragon fruit peel pectin may exhibit functional properties relevant to encapsulation systems. However, this potential is currently inferred from general pectin behavior rather than supported by direct experimental validation [74,107]. In composite delivery systems, pectin is often combined with other biopolymers such as chitosan, where chitosan is typically applied as an external coating or secondary layer, forming polyelectrolyte complexes that enhance acid resistance and structural stability of the capsules [107,109]. Therefore, further studies are required to establish the effectiveness of dragon fruit peel pectin as a probiotic delivery matrix and to clarify the relationship between its structure and encapsulation performance.

## 9. Synbiotic System Design: A Framework for Dragon Fruit Peel Pectin

### 9.1. The Dual-Role Design Principle and Its Structural Tension

The evidence reviewed in Section 3, Section 4, Section 5, Section 6, Section 7 and Section 8 suggests that dragon fruit peel pectin may theoretically fulfill both structural carrier and prebiotic substrate roles within a single polysaccharide architecture. However, it should be noted that these functions impose partially competing structural requirements. Carrier integrity in Ca^2+^-crosslinked systems is primarily dictated by DE, the distribution of de-esterified HG blocks, and GalA content, which collectively determine egg-box junction density and network rigidity. Low-DE pectins with contiguous free carboxyl groups have been reported to promote stronger Ca^2+^-mediated crosslinking due to enhanced junction-zone formation [110,111].

In contrast, HM pectins form gels through hydrogen bonding and hydrophobic interactions under conditions of low pH and high soluble solids content. While these gelation requirements are less compatible with probiotic viability during encapsulation, HM pectins may still contribute to synbiotic system design as fermentable substrates or as components of hybrid or layered delivery systems. Therefore, although LM-type pectins are generally more suitable as structural carriers, HM pectins may play a complementary role in synbiotic formulations depending on the targeted application [74,111].

Prebiotic functionality is associated with enrichment of RG-I domains and preservation of arabinan- and galactan-rich side chains, which have been shown to enhance fermentability and selectively stimulate probiotic growth [9,47]. As discussed in Section 7.4, enrichment of RG-I domains and preservation of arabinan- and galactan-rich side chains are associated with enhanced probiotic growth responses [9]. Because the extraction route strongly influences DE, MW, and RG-I preservation, processing conditions have been reported to influence structural characteristics and, consequently, functional behavior [66].

**Figure 2:**
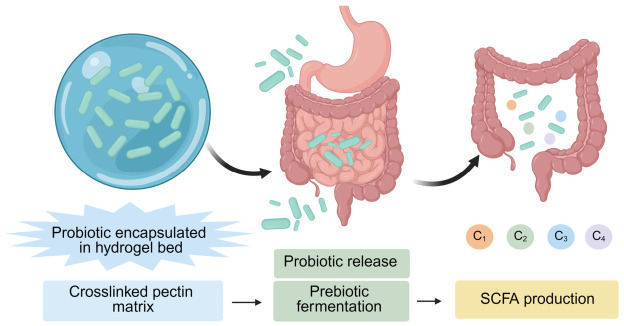
Synbiotic concept for dragon fruit peel pectin.

Collectively, these observations suggest the existence of a structural trade-off: features that favor Ca^2+^-mediated gel strength (e.g., low DE and extended HG blocks) are not identical to those associated with probiotic stimulation (e.g., RG-I branching density). It should be emphasized that this relationship is inferred from current evidence rather than directly demonstrated in dragon fruit peel pectin-specific synbiotic systems. Strategies such as controlled post-extraction de-esterification, composite or multilayer matrix architectures, and targeted structural modification have been reported in pectin-based systems [109,112], but their application to dragon fruit peel pectin remains to be systematically evaluated. (Figure 2 illustrates the synbiotic concept for dragon fruit peel pectin.)

### 9.2. GI Performance and Functional Integration

For synbiotic systems, effective performance requires protection of probiotic cells during gastric and small intestinal transit, together with the availability of fermentable substrates in the colon. Ca^2+^-crosslinked pectin hydrogels have been reported to exhibit pH-responsive behavior, remaining relatively stable under acidic gastric conditions while undergoing swelling or degradation under neutral to mildly alkaline intestinal conditions [111,112]. As described in Section 7.1, pectin is resistant to digestion in the upper GI and becomes available for microbial fermentation in the colon, with degradation behavior influenced by its structural features [99].

In addition, human clinical evidence indicates that dragon fruit-derived carbohydrates can modulate gut microbiota composition. A randomized, double-blind, placebo-controlled study demonstrated that DFO consumption influenced microbial populations and SCFA production in healthy adults [104]. However, it should be noted that these findings are based on low-MW oligosaccharides rather than intact dragon fruit peel pectin.

Within a synbiotic framework, the substrate component is ideally utilized by the co-administered microorganism [113]. In this context, RG-I-rich dragon fruit peel pectin may provide a fermentable substrate while simultaneously acting as a structural matrix. It is therefore reasonable to suggest that dragon fruit peel pectin could support both delivery and metabolic functions; however, this concept remains largely theoretical and has not yet been systematically demonstrated in integrated dragon fruit peel pectin-based systems.

## 10. Safety, Quality, and Regulatory Readiness

Dragon fruit peel pectin inherits a favorable regulatory foundation established for commercial pectin across major global jurisdictions. In the United States, pectin is recognized as a Generally Recognized As Safe (GRAS) substance under U.S. Food and Drug Administration (FDA) regulations (Title 21 of the Code of Federal Regulations [21 CFR] §184.1588) [114]. In the European Union (EU), pectin is approved as a food additive (E440) under Regulation (European Commission [EC]) 1333/2008 and has been comprehensively evaluated by the European Food Safety Authority (EFSA) [115,116]. In addition, pectin is permitted for use under the National Food Safety Standard for Food Additives (GB2760-2014) in China [117,118]. These existing regulatory frameworks provide a potentially advantageous starting point compared with entirely novel food ingredients that require de novo safety evaluation.

However, it should be noted that this regulatory applicability is conditional, as it applies specifically to pectins that meet defined compositional criteria, including a minimum GalA content of approximately 65%, as specified in international pectin standards (e.g., Codex Alimentarius and Joint Food and Agriculture Organization [FAO]/World Health Organization [WHO] Expert Committee on Food Additives [JECFA] specifications) [119]. In the case of dragon fruit peel pectin, reported GalA contents vary depending on extraction conditions, indicating that not all preparations would meet these regulatory thresholds [66]. Therefore, control of GalA content can be achieved through optimization of extraction conditions (pH, temperature, and time) and purification strategies to enrich HG-rich fractions [66,111,120], combined with routine compositional analysis to ensure batch consistency.

Beyond compositional requirements, the 2021 re-evaluation by the European Food Safety Authority Panel on Food Additives and Flavourings (EFSA FAF Panel) identified several safety data gaps for pectin (E440), including the need for updated microbiological specifications and revised limits for heavy metals such as arsenic, lead, mercury, and cadmium, as well as the proposed inclusion of aluminum [116]. These considerations may be particularly relevant for non-conventional pectin sources such as dragon fruit peel.

It should also be noted that published studies on dragon fruit peel pectin have not systematically reported heavy metal content, microbiological quality, or residual solvent levels, indicating that the safety data required for regulatory evaluation remain limited.

With respect to health claims, no jurisdiction has approved a specific prebiotic claim for pectin. The closest available clinical evidence relates to DFO fractions rather than intact pectin polymers, suggesting potential but not yet providing direct support for dragon fruit peel pectin-specific claims [104].

Table 6 summarizes the regulatory status across jurisdictions, and Table 7 consolidates safety specifications, distinguishing what currently exists from what remains undefined for dragon fruit peel pectin.

## 11. Challenges, Research Gaps, and Future Perspectives

### 11.1. Structural Variability as a Literature Problem

The most persistent challenge in the dragon fruit peel pectin literature is not a scientific knowledge gap but a reporting consistency problem that makes the available knowledge difficult to synthesize and apply. In addition to methodological differences, biological variability arising from different dragon fruit varieties (e.g., red- and white-fleshed cultivars) and growing conditions may also contribute to the observed variation in GalA content and MW [66,74]. Differences in cell wall composition, pectin domain distribution, and maturity stage have been reported to influence pectin structure, including GalA proportion, DE, and molecular size [74,124]. However, the magnitude of variability reported across studies suggests that methodological differences remain the dominant source of inconsistency. As Table 3 demonstrates, GalA content spans 49–84%, DE ranges from below 30% to above 60%, and MW varies from 33.52 kDa to over 1000 kDa across published studies. The ranges are so wide that they encompass structurally distinct material classes, not simply natural biological variation.

A substantial proportion of this apparent variability is attributable to methodological heterogeneity rather than genuine source differences. For DE/DM, FTIR-based estimation can diverge from wet-chemistry titration/HPLC approaches depending on calibration and sample composition, so cross-study comparisons are not always equivalent [125]. For MW, SEC using conventional calibration is conformation-dependent, and absolute MW determination typically requires light scattering (e.g., SEC-MALS), so reported Mw can be biased when conformation differs across samples or methods [126]. For GalA/uronic acids, colorimetric uronic-acid assays (e.g., m-hydroxydiphenyl) reduce—but do not eliminate—interference risks under strong-acid conditions, while chromatographic methods directly quantify monosaccharides/uronic acids and improve specificity [127,128].

Critically, DB, which strongly influences Ca^2+^-mediated gel formation, is rarely reported in dragon fruit-peel pectin studies. Broader pectin research has demonstrated that methyl ester distribution pattern influence gel stiffness more directly than average DE alone [129,130]. Enzymatic fingerprinting using endo-polygalacturonase digestion followed by HPAEC-PAD mapping provides a validated approach for assessing blockiness and ester distribution [131]. The absence of consistent reporting for DB, acetylation degree, and domain distribution limits predictive linkage between structure and techno-functional performance across studies. Establishing minimum reporting standards, specifying analytical methodology for DE, MW (including calibration approach), GalA quantification, DB, and acetylation would substantially enhance reproducibility and enable robust meta-analysis of structure–function relationships.

### 11.2. Limited In Vivo and Clinical Validation

Current evidence for the prebiotic potential of dragon fruit peel pectin primarily derives from single-strain in vitro growth assays using isolated probiotic cultures [47]. In contrast, human randomized controlled trials in this area have largely evaluated dragon fruit-derived oligosaccharides rather than intact peel pectin polymer [104]. Within dietary fiber research more broadly, in vitro fecal batch fermentation using multi-donor human microbiota with SCFA quantification represents an established translational screening approach for assessing selective microbial utilization [132]. However, comprehensive fecal fermentation studies specifically evaluating intact dragon fruit peel pectin remain limited. Likewise, controlled animal feeding studies assessing purified dragon fruit peel pectin with microbiome profiling and intestinal barrier endpoints are sparse in the current literature. Encapsulation of probiotic strains within pectin-based matrices followed by sequential simulated GI digestion is an established synbiotic validation methodology [133]. Nevertheless, dragon fruit peel pectin-specific probiotic delivery systems have not been extensively characterized using these standardized approaches. According to the International Scientific Association for Probiotics and Prebiotics (ISAPP) consensus definition, a prebiotic must be selectively utilized by host microorganisms and confer a health benefit. For intact dragon fruit peel pectin, demonstration of selective microbiota modulation and associated [121] health-relevant outcomes remain an important priority before formal prebiotic classification can be firmly established.

### 11.3. Scale-Up, Techno-Economic, and Life Cycle Considerations

Dragon fruit peel is a geographically concentrated and seasonally variable biomass stream, unlike citrus peel, which benefits from globally established year-round industrial processing infrastructure. Extraction yields from dragon fruit peel are strongly method-dependent and vary considerably across reported extraction techniques [27]. Due to its high betalain pigment content, additional clarification or decolorization steps may be required for certain food applications, potentially increasing processing complexity [35]. From a valorization perspective, betalains generally represent a higher-value product due to their application as natural colorants and antioxidants in food and cosmetic industries, whereas pectin is typically a bulk functional ingredient with lower unit value but larger market volume [74,134]. Therefore, integrated biorefinery strategies that enable sequential recovery of betalains prior to pectin extraction may offer a more economically advantageous approach, allowing co-production of high-value pigments and functional polysaccharides from the same biomass stream [135]. A life cycle assessment (LCA) of conventional pectin production from fruit waste has estimated a base-case environmental impact of approximately 9.69 kg CO_2_-equivalent per kg pectin produced, with acid use contributing substantially to the overall burden [136]. Compared to conventional acid-assisted heat procedures, pilot-scale acid-free microwave-assisted extraction has been demonstrated to significantly lower environmental impacts [137].

A thorough LCA contrasting commercial citrus pectin manufacturing with dragon fruit peel pectin extraction methods (acid extraction, subcritical water extraction, ultrasound-assisted extraction, and microwave-assisted extraction) has not yet been published. Assessments of sustainability and commercialisation viability may be greatly impacted by the inclusion of betalain co-valorization in techno-economic modeling.

### 11.4. Integrated Research Roadmap

Advancing dragon fruit peel pectin from structural characterization toward validated synbiotic application requires a sequential and integrated research framework. First, systematic characterization of methyl ester distribution (DB) across extraction methods should be conducted using enzymatic fingerprinting and chromatographic mapping, as established in broader pectin structural analysis [130,131]. Such data would enable rational prediction of Ca^2+^-mediated gel strength and encapsulation potential. Second, structurally distinct dragon fruit peel pectin variants should be evaluated using multi-donor in vitro fecal batch fermentation models with 16S rRNA profiling and targeted SCFA quantification, consistent with established translational screening approaches [132]. Third, a standardized Ca^2+^-crosslinked bead model system incorporating structurally characterized dragon fruit peel pectin should be developed and subjected to sequential simulated GI transit assays to quantitatively determine viability retention, matrix stability, and release kinetics under controlled conditions, following established synbiotic evaluation protocols [133]. These *in vitro* findings should then inform controlled murine feeding studies incorporating microbiome composition, SCFA production, and intestinal barrier markers to establish biological relevance. In addition, the potential role of HM pectins should be considered within the synbiotic design framework. Although HM pectins are less suitable for Ca^2+^-mediated encapsulation due to their requirement for low pH and high soluble solids for gelation [74,111], they may still contribute as fermentable substrates or as components in hybrid or multilayer delivery systems [107,109]. Exploring combinations of HM and LM pectin fractions, or their integration into composite matrices, may provide a strategy to balance structural functionality and prebiotic activity in future system designs. Finally, parallel life cycle and techno-economic assessments comparing dragon fruit peel extraction routes with conventional citrus pectin production would provide the sustainability and commercialization evidence base necessary for industrial translation [136,137].

## 12. Conclusions

Pectin from dragon fruit peels is a sustainable and structurally adjustable substitute for traditional pectin sources. Its extraction-dependent variability allows for customized manipulation of techno-functional and biological features, but it hampers direct substitution in standardized applications. Its promise as a fermentable dietary fiber and a probiotic delivery matrix is supported by evidence, providing a basis for the design of synbiotic systems. However, prior to commercial translation, harmonized structural characterization, *in vivo* validation, and regulatory compliance assessment are necessary. Dragon fruit peel pectin may become a scientifically validated functional element in sustainable food innovation methods with thorough standardization and translational research.

## Figures and Tables

**Table 1:** Compositional ranges and physicochemical characteristics of dragon fruit peel, citrus and apple pectins.

Parameter	Dragon Fruit Peel Pectin	Citrus Pectin (Commercial)	Apple Pectin (Commercial)	Reference
GalA	GalA-dominant; ≈39–87% depending on extraction [31,46]	≥65% (E440)	≥65% (E440)	[27,57,63]
DE, %	Frequently within a 30–60% extraction-dependent DE window, near the transition between LM and HM pectins [31,58]	HM (>50%) or LM (<50%)	HM or LM classification	[31,53,57,58]
MW (kDa)	Hundreds to >1000 kDa (method-dependent) [27,31]	Controlled high MW for gel strength	Moderate–high controlled MW	[27,31,53]
HG/RG-I composition	HG + substantial RG-I domains [27]	HG-dominant	HG with some RG-I	[27,28,53]
Ara/Rha and related neutral-sugar ratios	Variable Ara/Rha, (Ara + Gal)/Rha ratios [27]	Lower neutral sugar ratios	Moderate neutral sugar contents and branching	[27,28,53]
Co-extracted compounds	Phenolics, betalains often retained [44]	Highly purified	Highly purified	[31,44,53]
Gelation mechanism	DE-dependent gelation: LM → Ca^2+^ “egg-box”; HM → sugar–acid (inferred from DE range) [27,55]	HM: sugar–acid; LM: Ca^2+^ egg-box	HM: sugar–acid; LM: Ca^2+^ egg-box	[27,53,55]
Industrial standardization	No harmonized framework	E440 fully standardized	E440 fully standardized	[16,54,63]
Application readiness	Emerging: films, emulsifiers	Jams, dairy, confectionery	Fruit prep, dairy	[31,36,53]

**Table 2:** Structural and functional profiles of dragon fruit peel pectins for different extraction strategies.

Extraction Method	Structural/Extraction Trend	Functional Implication	Reference
Hot Acid/Conventional	Baseline pectin extraction; used for comparison in many studies	Reference method for structural comparison	[57]
UAE	Enhances extraction yield vs. conventional; potential changes in polymer structure	Increased yield; altered viscosity/structural features	[45]
MAE	Higher extraction efficiency; varying intensity affects yield and polymer properties	Higher yield with possible degradation at high power	[69]
SCWE	Improved yield vs. acid extraction; produces LM pectin with distinct film properties	Leads to distinctive functional properties like film thermal stability	[66]
Microwave–Ultrasound-assisted extraction (MUAE)/NADES Combined Approaches	Sequential ultrasound–microwave/NADES can lead to higher extraction efficiency and tailored physicochemical traits	Potential for greener, tunable pectin profiles	[58,71]

**Table 3:** Structural features of dragon fruit peel pectins: reported parameter ranges, analytical methods, and functional implications.

Structural Parameter	Reported Range	Analytical Method(s)	Functional Implication	Reference
GalA, %	39–87%	High-Performance Anion-Exchange Chromatography with Pulsed Amperometric Detection (HPAEC-PAD); colorimetric uronic acid assay	Backbone purity; gelling capacity; prebiotic substrate quality	[27,31,46]
Rha, %	1–9%	HPAEC-PAD	RG-I backbone indicator; influences chain flexibility	[27,47]
Ara, %	3–15%	HPAEC-PAD	Side chain density; fermentability by gut microbiota	[27,47]
Gal, %	3–12%	HPAEC-PAD	AG side chain contribution; viscosity and prebiotic effects	[27,47]
(Ara + Gal)/Rha ratio	2–8+	Calculated from HPAEC-PAD data	Proxy for RG-I side chain substitution density	[47]
DE, %	30–60%	FTIR (semi-quantitative); titrimetric method	Gelation mechanism (Ca^2+^ vs. sugar–acid); solubility; emulsification	[28,29,31,58]
Acetylation (acetyl content)	Rarely quantified; generally low	NMR; colorimetric	Influences gel texture and water-holding; underreported	[48]
Weight-average MW, kDa	33–1181 kDa	Size Exclusion Chromatography with Refractive Index detection (SEC-RI) or SEC-MALS	Viscosity; gel strength; fermentation rate; encapsulation matrix properties	[28,29,31]
Dispersity	Polydisperse	SEC	Indicates processing heterogeneity; affects functional consistency	[52]
RG-I side chain type	Arabinogalactan AG type inferred	Methylation–Gas Chromatography–Mass Spectrometry (GC-MS); NMR (HSQC)	Fermentability profile; bifidogenic/butyrogenic potential	[47]

**Table 4:** Structure–function mapping for dragon fruit peel pectins.

Functional Property	Primary Structural Drivers	Mechanistic Basis	Reference
Solubility	DE, charge, hydration; GalA content	Electrostatic hydration vs. methyl ester shielding	[24,45,58]
Viscosity	MW (primary); RG-I side chains (secondary)	Chain entanglement; hydrodynamic volume	[24,27,84]
Gelation	DE selector; GalA DB; MW strength	Ca^2+^ egg-box vs. acid–sugar H-bonding	[28,57]
Emulsification	MW thickness; RG-I amphiphilicity; co-extracts	Steric + interfacial + complexation	[31,52,66]
WHC	DE network; RG-I branching; MW extent	Hydration surface maximization	[27,57,69]
OHC	DE hydrophobicity; co-extractives	Hydrophobic domains in hydrophilic matrix	[31,45,84]

**Table 5:** Studies on the synbiotic relevance of dragon fruit-derived pectic polysaccharides and oligosaccharides.

Pectin/Substrate	RG-I Richness	Model	SCFA Results	Microbial Shift	Synbiotic Relevance	Reference
Red dragon fruit peel pectin obtained via three extraction methods (citric acid, ultrasonic, and hot water extraction)	RG-I 66.59 mol%; (Ara + Gal)/Rha = 1.25 (citric acid extract)	In vitro single-strain (*B. animalis* subsp. *lactis* 3296)	Not quantified (growth assay)	Citric acid extract showed greater promotion of *B. animalis* proliferation than ultrasonic and hot water extracts; RG-I side chain density identified as the key driver	Extraction-tunable prebiotic potency; RG-I side chains are the active structural determinant	[47]
Purified RG-I domain from red dragon fruit pectin; isolated by gel purification + enzyme digestion	RG-I backbone; branches: 1,4-linked galactose (galactan type I)	In vitro single-strain (*B. animalis* subsp. *lactis* 3296)	Not quantified	RG-I galactan branches drive *B. animalis* proliferation; promotes β-D-galactosidase production	Galactan side chains identified as key prebiotic components; preservation during extraction is essential for synbiotic design	[9]
DFO; fructan-type, low MW; from whole dragon fruit	Low MW oligosaccharide fraction; structurally related to pectic side chain fragments	Randomized controlled trial (RCT): *n* = 107 healthy adults; 4 and 8 g/day vs. placebo; 4 weeks	SCFAs inferred from microbiota shifts; acetate, propionate, butyrate noted as fermentation end-products	8 g/day: *Bifidobacterium* 8.41%, *Faecalibacterium* 1.99%, *E. coli* 8.44%; 4 g/day: Immunoglobulin A (IgA) 11.31 mg/dL (+10.95%)	Strongest clinical evidence for dragon fruit polysaccharide prebiotic efficacy; dose–response established; IgA endpoint supports mucosal immunity argument for synbiotic formulations	[104]
DFO; white-fleshed *H. undatus*	Low MW fructan-type oligosaccharide	In vivo rat model + 3-stage continuous colon system; safety + efficacy assessed	Prebiotic index (PI) = 0.41 from fecal fermentation; SCFA production inferred	Fecal *Bifidobacterium* ↑, *Lactobacillus* ↑; *Bacteroides* ↓, Clostridia ↓; plasma IgA and Immunoglobulin G (IgG) ↑; colon histology normal	Supports safety profile for synbiotic ingredient use	[105]

Note: ↑ indicates increase; ↓ indicates decrease.

**Table 6:** Regulatory status of pectin and implications for dragon fruit peel pectin across major jurisdictions.

Jurisdiction	Regulatory Framework	Current Pectin Status	Key Compositional Threshold	Dietary Fiber/Prebiotic Claim	Implication for Dragon Fruit Peel Pectin	Reference
EU	Regulation (EC) 1333/2008; E440i/E440ii	Approved food additive	GalA ≥ 65%; LM/HM classification	No authorized EU prebiotic health claim; dietary fiber declaration permitted	GalA < 65% does not meet E440; improve via extraction optimization and purification (HG enrichment), or reclassify as dietary fiber.	[115,116]
United States	FDA Title 21 of the Code of Federal Regulations (21 CFR); GRAS	GRAS; explicitly listed as dietary fiber on FDA Nutrition Facts Label	No minimum GalA for GRAS; dietary fiber requires demonstrated physiological benefit	Structure–function claims for gut microbiota modulation permissible without premarket approval; disclaimer required	GRAS broadly applicable; dietary fiber labeling achievable; prebiotic structure–function claim supportable with RCT evidence	[114]
China	GB 2760-2014 Food Additive Standard	Approved; no maximum usage level	GalA not explicitly specified	Functional food health claims via National Medical Products Administration (NMPA) registration pathway	Broad approval applicable; functional food claim requires NMPA registration, lengthy and costly	[117]
Global prebiotic claim	No harmonized international framework	No jurisdiction has approved a pectin-specific prebiotic health claim	Clinical RCT in humans required for substantiation in all jurisdictions	Intact pectin polymer RCT absent	Full prebiotic claim not currently supportable in any jurisdiction without additional intact-pectin clinical data	[104,121]

**Table 7:** Safety specifications, contaminant limits, and standardization requirements for dragon fruit peel pectin.

Parameter	Current E440/Regulatory Specification	Status for Dragon Fruit Peel Pectin	Gap/Action Required	Reference
GalA content (min.)	≥65% (E440/Codex/JECFA)	39–87% reported; GalA > 65% achieved under optimized extraction conditions (e.g., controlled pH, temperature, and extraction method)	Preparations below 65% require reclassification or purification optimization	[27,115,116]
DE classification	LM (<50%) or HM (>50%) declared	30–60%	No harmonized DE reporting standard; classification ambiguity at borderline values	[47,66]
MW	Not specified in E440 framework	33–1200 kDa; SEC vs. SEC-MALS variation	MW range and dispersity specifications absent; critical for encapsulation and gelation consistency	[27]
Heavy metals—Arsenic	≤3 mg/kg (EU); EFSA 2021 recommends lowering	No published screening data	Systematic heavy metal screening required; soil contamination risk in Southeast Asian/Latin American cultivation systems	[116]
Heavy metals—Lead	≤5 mg/kg (EU); revision proposed	No published data	Same as arsenic above	[116]
Heavy metals—Mercury	≤1 mg/kg (EU)	No published data	Same as arsenic above	[116]
Heavy metals—Cadmium	≤1 mg/kg (EU)	No published data	Same as arsenic above	[116]
Heavy metals—Aluminum	Not in current E440 spec; EFSA 2021 recommends addition	No published data	New specification anticipated; dragon fruit peel data entirely absent	[116]
Microbiological-*Salmonella*	Absence required (EFSA recommendation; not yet in EU 231/2012)	No published microbiological data	Formal microbiological testing protocol required before any regulatory submission	[116]
Microbiological *E. coli*	Absence required (EFSA recommendation)	No published data	Same as *Salmonella* above	[116]
Betalain/pigment content	No specification; colorant declaration may apply above threshold	Retained in less-purified fractions; concentration unreported systematically	Quantification needed; if above declaration thresholds, additional labeling required	[31]
Allergenicity	Pectin not classified as allergen in any major framework	No allergenicity concern identified	Confirm via standard allergen cross-reactivity assessment; no action anticipated	[115]
Acetylation	Not specified in E440 framework	Rarely quantified in dragon fruit literature (Section 4.3)	Recommend inclusion in minimum reporting standard; influences gel texture and enzymatic susceptibility	[55]
DB	Not specified in any regulatory framework	Not reported for any dragon fruit peel pectin preparation	Critical for encapsulation performance prediction; should be minimum characterization standard in future studies	[122,123]
Industrial standardization	E440 fully standardized for citrus/apple pectin	No harmonized framework exists for dragon fruit peel pectin	Voluntary industry standard or formal food additive petition required before commercial adoption in EU/US	[115,117]

## Data Availability

No new data were created or analyzed in this study. Data sharing is not applicable to this article.

## Abbreviations

The following abbreviations are used in this manuscript:

Ara	Arabinose.
AG	Arabinogalactan.
CFR	Code of Federal Regulations.
CFU	Colony-forming units.
DB	Degree of blockiness.
DE	Degree of esterification.
DES	Deep eutectic solvent.
DFO	Dragon fruit oligosaccharides.
EFSA	European Food Safety Authority.
EC	European Commission.
EU	European Union.
FAO	Food and Agriculture Organization.
FDA	U.S. Food and Drug Administration.
FTIR	Fourier Transform Infrared Spectroscopy.
Gal	Galactose.
GalA	Galacturonic acid.
GC-MS	Gas Chromatography–Mass Spectrometry.
GI	Gastrointestinal.
GRAS	Generally recognized as safe.
HG	Homogalacturonan.
HM	High-methoxyl (pectin).
HPAEC-PAD	High-Performance Anion-Exchange Chromatography with Pulsed Amperometric Detection.
HPLC	High-Pressure Liquid Chromatography.
HSQC	Heteronuclear Single Quantum Coherence Spectroscopy.
IgA	Immunoglobulin A.
IgG	Immunoglobulin B.
ISAPP	International Scientific Association for Probiotics and Prebiotics.
JECFA	Joint FAO/WHO Expert Committee on Food Additives.
LCA	Life Cycle Assessment.
LM	Low-methoxyl (pectin).
MAE	Microwave-assisted extraction.
MALS	Multi-angle light scattering.
MUAE	Microwave–Ultrasound-assisted extraction.
MW	Molecular weight.
NMPA	National Medical Products Administration.
NMR	Nuclear Magnetic Resonance Spectroscopy.
NADES	Natural deep eutectic solvent.
OHC	Oil-holding capacity.
PDI	Polydispersity index.
PI	Prebiotic Index.
PME	Pectin methylesterase.
RCT	Randomized Controlled Trial.
RG-I	Rhamnogalacturonan-I.
RG-II	Rhamnogalacturonan-II.
Rha	Rhamnose.
SCFA	Short-chain fatty acids.
SWE/SCWE	Subcritical water extraction.
SEC	Size-exclusion chromatography.
SEC-RI	Size Exclusion Chromatography with Refractive Index detection.
UAE	Ultrasound-assisted extraction.
WHC	Water-holding capacity.
WHO	World Health Organization.
